# Differences among Research Domain Criteria score trajectories by Diagnostic and Statistical Manual categorical diagnosis during inpatient hospitalization

**DOI:** 10.1371/journal.pone.0237698

**Published:** 2020-08-25

**Authors:** Thomas H. McCoy, Amelia M. Pellegrini, Roy H. Perlis

**Affiliations:** 1 Center for Quantitative Health, Massachusetts General Hospital, Boston, MA, United States of America; 2 Department of Psychiatry, Harvard Medical School, Boston, MA, United States of America; 3 Department of Medicine, Harvard Medical School, Boston, MA, United States of America; University of Toronto, CANADA

## Abstract

With brief psychiatric hospitalizations, the extent to which symptoms change is rarely characterized. We sought to understand symptomatic changes across Research Domain Criteria (RDoC) dimensions, and the extent to which such improvement might be associated with risk for readmission. We identified 3,634 individuals with 4,713 hospital admissions to the psychiatric inpatient unit of a large academic medical center between 2010 and 2015. We applied a natural language processing tool to extract estimates of the five RDoC domains to the admission note and discharge summary and calculated the change in each domain. We examined the extent to which symptom domains changed during admission, and their relationship to baseline clinical and sociodemographic features, using linear regression. Symptomatic worsening was rare in the negative valence (0.4%) and positive valence (5.1%) domains, but more common in cognition (25.8%). Most diagnoses exhibited improvement in negative valence, which was associated with significant reduction in readmission risk. Despite generally brief hospital stays, we detected reduction across multiple symptom domains, with greatest improvement in negative symptoms, and greatest probability of worsening in cognitive symptoms. This approach should facilitate investigations of other features or interventions which may influence pace of clinical improvement.

## Introduction

In an era of brief psychiatric hospitalizations, the extent to which individuals achieve symptomatic improvement is not well characterized. On one Italian inpatient unit with an average length of stay of 5.7 days, for example, only 13.6% of individuals achieved 'clinically meaningful change' [1]. The modest improvement is not wholly attributable to shorter lengths of stays, as older studies indicated substantial variability in degree of improvement [2]. In general, the aim of hospitalization is not recovery, but sufficient stabilization for safe management at a lower level of care. Such stabilization often entails decrease in a single particularly acute symptom that precipitated hospitalization, such as suicidality or aggressive behavior, rather than modest improvements in a wide range of symptom domains. As such, while many studies focus on changes in syndrome-specific symptomatology, like depression [3] or psychosis [4], few have examined psychiatric symptomatology from a broader transdiagnostic perspective, nor sought to investigate whether some symptoms actually worsen during hospitalization.

Another rarely studied question is whether such inpatient improvement actually matters in longer-term outcomes; that is, does more rapid improvement aid in identifying a population at lower readmission risk? Or, conversely, might such improvement instead represent a fluctuating illness course, such that readmission is actually more likely compared to individuals with more chronic symptomatology? In one study of inpatients receiving psychotherapy, rapid improvement in a general measure of psychopathology indicated better outcomes at 6 months[5].

Recognizing the difficulty in extrapolating from this sparse literature to a modern inpatient psychiatric unit, we applied a recently-validated method for characterizing symptom burden across five dimensions approximating the National Institute of Mental Health (NIMH) Research Domain Criteria (RDoC) [6–9]. The NIMH RDoC system is a framework for organizing neuropsychiatric symptoms into five domains which are rooted in neuroscience and independent of conventional diagnostic labels [10–12]. Estimates of the RDoC domains based on clinical documentation have shown associations with healthcare utilization and death by suicide and have been applied successfully in a genome-wide association study [13–15]. Given the emerging importance of RDoC and the uncertainty of current clinical trajectories of hospitalized patients, we sought to understand both the extent to which symptom domains change for better or worse, and then whether these changes are associated with differential risk for readmission.

## Materials and methods

### Study cohort and variables

We investigated a retrospective cohort drawn from an inpatient general psychiatry unit in a large academic medical center. Electronic health records were used to identify the admission notes and discharge summaries (which exclude admission notes) for all inpatient psychiatric admissions between 2010 and 2015, as well as sociodemographic features, including age at admission, sex, race, and insurance type (public versus private payer). As a measure of overall comorbidity, Charlson comorbidity index was calculated based on billing codes prior to admission and the primary diagnosis for each admission was categorized based on ICD9 codes. Registration data was used to calculate length of hospital admission in days. A data mart with all data was generated using Informatics for Integrating Biology and the Bedside, or i2b2, server software [16,17].

The institutional review board of Partners HealthCare reviewed and approved the study protocol; the requirement for informed consent was waived as a retrospective health care utilization study with no patient contact.

### Calculation of estimated RDoC (eRDoC) scores by natural language processing

We have previously reported a method for deriving estimates of the five RDoC domains from narrative clinical notes [8] and made this tool available as open-source software [18]. This freely available tool draws on expert-curated token lists, expanded via unsupervised machine learning to identify synonyms of those terms which occur in clinical documentation. These final lists are then converted into scores as the proportion of terms appearing in any given note. For further details of the scoring method and validation see McCoy, Yu et al., 2018 [8,15]. In the present study, estimated RDoC *difference* scores were calculated as the difference between the discharge summary domain estimate and the admission note domain estimate. For example, a patient with an eRDoC negative valence estimate of 0.5 on admission and 0.1 on discharge would have a difference score of -0.4.

### Outcomes

Time to hospital readmission was determined by identifying the date of rehospitalization documented in the electronic health record, where available. Individuals lost to follow-up within 90 days—i.e., those for whom there were no documented facts (ICD9 diagnosis or CPT codes) at or beyond 90 days—were excluded from these analyses.

### Analysis

As we sought to understand the relationship between clinical features and admissions, the primary unit of analysis was admission, recognizing that such admissions could be clustered within individuals (i.e., an individual might have multiple admissions). We first characterized individual (subject) level difference from admission to discharge in estimated symptoms within domain to describe the change during hospitalization. To characterize changes over the course of a hospital encounter within a given domain by diagnostic groups, we used one sample t-tests of each domain difference score within diagnostic group. We then identified the subset of encounters associated with worsening in each domain, defined a priori as at least a 1 standard deviation *increase* in domain score between admission and discharge. Finally, we used analysis of variance to test for diagnostic category group differences in change in estimated domain symptom burden over the course of hospitalization. Where omnibus test was nominally significant at p<0.05, Tukey’s HSD test was used to examine pairwise comparisons while controlling type 1 error. We also sought to understand the association between change score and 90-day readmission, using mixed effects logistic regression (to address admissions nested within patients, where available), among those patients who had adequate follow up to observe the outcome. This analysis was conducted as both a univariate (crude) analysis and as a multivariate analysis adjusting for age, race (white vs nonwhite), sex, insurance type (presence/absence of public insurance), length of hospital stay, and age-adjusted Charlson comorbidity index, consistent with our prior work [13]. These covariates were included to adjust for potential confounding effects, rather than to examine independent effects.

## Results

The full cohort (Table 1, top) included 4,713 admissions for 3,634 individuals; the mean age was 45.0 (SD 16.6) years, and admissions were 49% male, 61% publicly insured, and 72% white. The most common primary diagnosis was major depressive disorder (n = 1,021, or 42.0%) followed by psychotic disorder (n = 775, or 31.9%), bipolar depression (n = 265, or 10.9%), and bipolar mania/mixed state (n = 168, or 6.9%). All but 63 (1.3%) had adequate follow-up for subsequent inclusion in the 90-day readmission analysis.

**Table 1:** Characteristics of all admissions.

Total Admissions (n)	4713
	**Mean (SD)**
Age at Discharge (years)	44.97 (16.64)
Length of Stay (days)	9.95 (8.89)
log(Age Adjusted Charlson Comorbidity Index)	0.68 (0.77)
	**n (%)**
Sex (male)	2327 (0.49)
Race (white)	3412 (0.72)
Publicly Insured	2873 (0.61)
*Readmission Outcome*	
Inadequate follow-up	63 (1.3)
Readmitted	1,639 (34.8)
*Primary Diagnosis*	
MDD	1021 (42.0)
Psychosis	775 (31.9)
BPAD-D	265 (10.9)
BPAD-M	168 (6.9)
Substance	99 (4.1)
Anxiety	63 (2.6)
PTSD	42 (1.7)
*Worsening by RDoC Domain*	
Negative Domain	21 (0.4)
Positive Domain	241 (5.1)
Cognitive Domain	1216 (25.8)
Social Domain	594 (12.6)
Arousal and Regulatory Domain	857 (18.2)

MDD = major depressive disorder

BPAD-D = bipolar affective disorder, depression

BPAD-M = bipolar affective disorder, mania/mixed

PTSD = post-traumatic stress disorder

RDoC = Research Domain Criteria

While the majority of individuals exhibited improvement, a subset worsened in each domain (Table 1, bottom), ranging from 21/4,713 (0.4%) for negative valence and 241/4,713 (5.1%) for positive valence to 1,216/4,713 (25.8%) for cognition.

**Fig 1:**
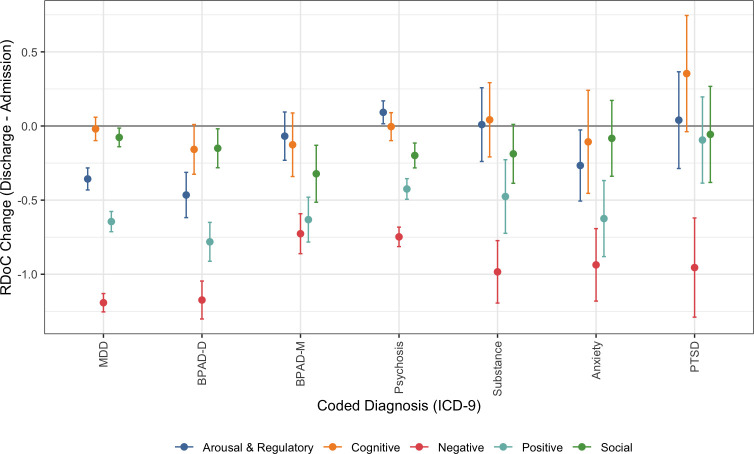
Average changes in estimated Research Domain Criteria scores between admission and discharge by ICD9 categorical diagnosis. Bars indicate 95% confidence intervals. MDD = major depressive disorder; BPAD-D = bipolar affective disorder, depression; BPAD-M = bipolar affective disorder, mania/mixed; PTSD = post-traumatic stress disorder.

The within diagnosis average of individual eRDoC difference scores for each domain are illustrated in Fig 1 (for numeric values, see S1 Table). Fig 1 illustrates the overall pattern of change, with negative symptoms significantly declining between admission and discharge (i.e., the confidence interval does not include zero) regardless of diagnostic category and cognitive symptoms failing to change significantly across all categories. Other domains were more variable across diagnostic categories with positive valence the second most frequently-improved (i.e., reduction between admission and discharge was observed in all diagnostic categories except PTSD [S1 Table]).

Turning from comparisons within diagnostic category (i.e., for a given diagnosis, what domains change the most) to those across diagnostic category (i.e., which domains change differentially by diagnosis), the average change in negative [F(6) = 18.582, p < .00001], positive [F(6) = 6.643, p < .00001], and arousal [F(6) = 14.679, p < .00001] eRDoC score over the course of admission differed significantly by ICD9 diagnostic category, whereas neither social [F(6) = 1.781, p = 0.10] nor cognitive [F(6) = 1.293, p = 0.26] eRDoC score did so. Diagnostic groups which differed significantly in *post hoc* pairwise testing of negative, positive, and arousal domain score main effects are shown in S2 Table.

**Fig 2:**
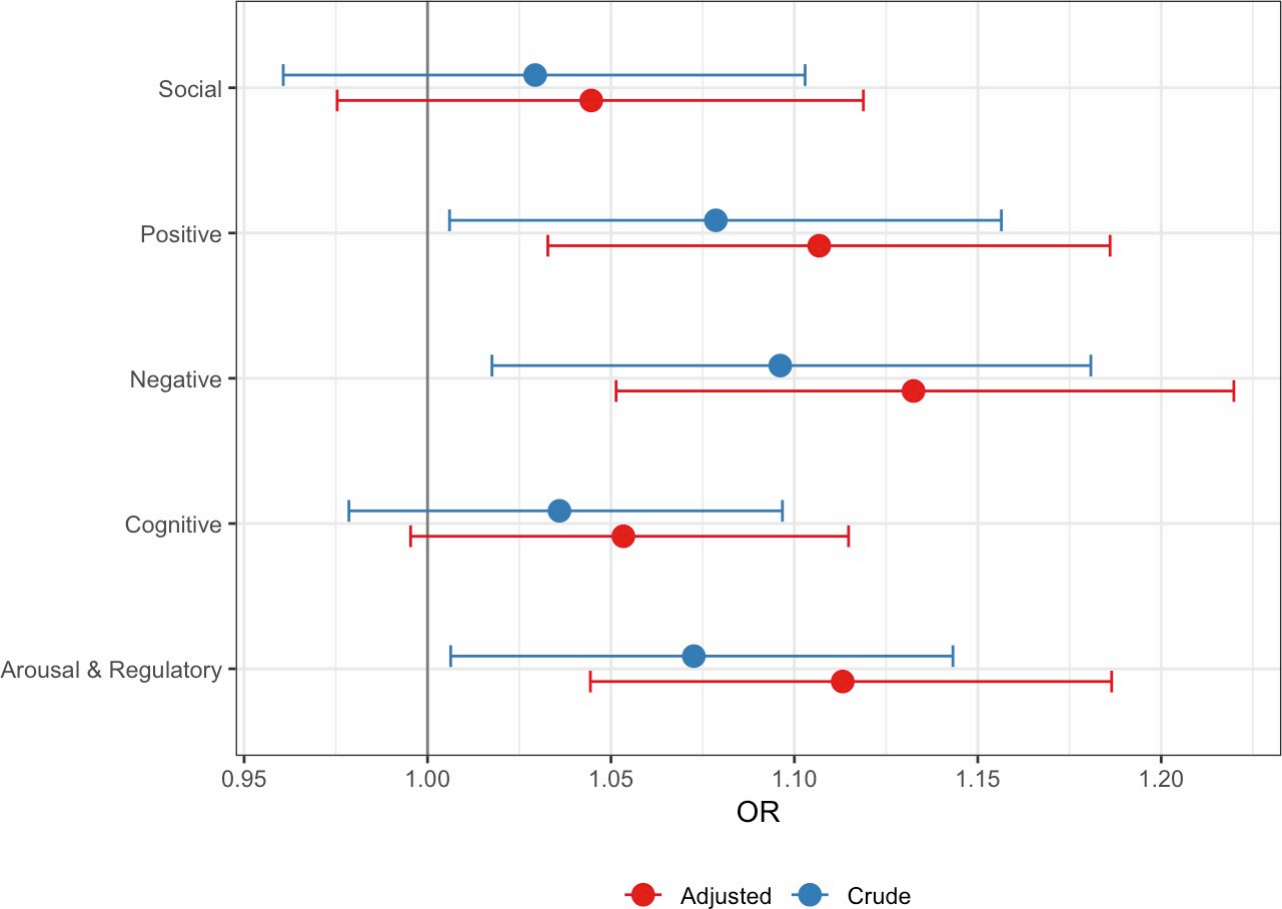
Crude and adjusted odds ratios (OR) for 90-day readmission risk, by change in estimated Research Domain Criteria score between discharge and admission.

Readmission within 90 days of hospital discharge occurred following 1,639 (34.8%) of the discharges. Of the five RDoC domains, change in positive valence, negative valence, as well as in arousal and regulatory process were all associated with early readmission in both crude and adjusted mixed effects logistic regression models (Table 2). Social process and cognitive symptom change were not significantly associated with 90-day regardless of covariates. To illustrate comparison across domains Fig 2 plots odds ratios and 95% confidence intervals from the logistic regression models predicting odds of 90-day readmission for changes in individual eRDoC domains, unadjusted and then after adjustment for sociodemographic and clinical features as shown in Table 2. Negative valence exhibited the strongest association with 90-day readmission [crude odds ratio 1.05–1.22] such that a patient who had a 1-point *increase* in eRDoC negative valence score between admission and discharge had ~13% greater odds of readmission.

**Table 2:** Crude and adjusted odds ratios (OR) for 90-day readmission risk, by change in estimated Research Domain Criteria score between admission and discharge.

Domain	Crude OR	[95% Conf. Int]	Adjusted OR*	[95% Conf. Int]
** Arousal & regulatory**	1.113	[1.044–1.186]	1.073	[1.006–1.143]
** Cognitive**	1.053	[0.995–1.115]	1.036	[0.979–1.097]
** Negative**	1.132	[1.051–1.22]	1.096	[1.018–1.181]
** Positive**	1.107	[1.033–1.186]	1.079	[1.006–1.156]
** Social**	1.045	[0.975–1.119]	1.029	[0.961–1.103]

*Adjusted odds ratios are adjusted for sex, race, age, Charlson, insurance type, and length of stay.

## Discussion

In this study of transdiagnostic symptom change during inpatient admission, we identified distinct patterns of association with individual estimated RDoC domains. Nearly all hospitalized patients across disorders experienced improvement in the negative valence domain, and such improvement was associated with reduction in readmission risk. A prior study of brief hospital stays found that only a minority—less than 15%—were associated with clinically meaningful improvement [1]. Our results suggest that most patients achieve some improvement in negative valence, which is meaningful insofar as it associates with reduced readmission risk. A prior study using a brief depression screen during hospitalization (while not a change score per se) likewise suggested that less improvement is associated with readmission [19].

We further found that worsening in cognitive domain scores was detectable in about one-fourth of hospitalized patients, but that short-term cognitive change did not associate with likelihood of readmission. Our findings raise the important question of why a subset of hospitalized psychiatric patients might experience short-term worsening in cognition, identifying a rarely-studied but potentially informative subgroup which may give insight into both important connections among mood and memory disorders and into the utility of RDoC based cognitive screening.[20,21] Further study of those who worsen, in some regards, is an important further direction for transdiagnostic research building on prior work within diagnostic groups. [3,22–24]

We note multiple limitations in interpreting our results. First, absent structured scales, we cannot relate these findings to standard measures beyond our prior validation efforts [13–15]. In particular, it is possible that our observation of modest change in cognitive symptoms reflects a lack of sensitivity for such change, compared to (for example) neurocognitive measures. On the other hand, we would underscore that rating scales are not a standard aspect of care in most clinical settings, so non-research measures may be more feasible for large-scale application. Second, some of the change observed in a given domain may reflect changes in words used in admission compared to discharge notes–in the former case, a note may seek to justify admission, while in the latter a note may seek to justify discharge. In general, this expectation would likely bias us toward greater improvement (if symptoms are minimized for discharge) but does not preclude examining associations with outcomes or comparisons across diagnoses. Third, these findings pertain to a single hospital unit; while we have previously shown them to be valid across populations, the ability to detect symptom change merits study in other populations. We also note that the tool used in this analysis does not include the newly added sixth RDoC domain, which focuses on sensorimotor symptoms.[25] The study is strengthened by the use of a large general psychiatric cohort cared for in a comprehensive medical system and use of a transdiagnostic approach—estimating RDoC scores from clinical text–which has been studied in both clinical and translational contexts and is freely available. In combination this study combines the strength of high throughput RDoC phenotyping and a diverse clinical cohort to directly address the RDoC aim of look across, and sub-dividing within current diagnostic categories.[26,27]

Taken together, our results showing variability in RDoC domain trajectory within and across diagnosis and subsequent association with clinical utilization illustrate the application of a transdiagnostic approach to understand not only dimensions of psychopathology, but patterns of change, that is trajectories, in these symptoms over time. They suggest that even as some domains improve, others may remain the same or worsen during a hospital stay, and that such improvement patterns associate with posthospital outcomes. This evidence of clinical outcome variability based on the specific composition of transdiagnostic neuropsychiatric constructs, and changes among those symptom domains, is a critical step toward realizing the goal of psychiatric precision medicine.[11,24,28–30]

The study results also show the ability of a natural language processing tool to detect magnitude of clinical improvement, even in the absence of formal rating scales. This approach does not replace such scales but acknowledges that, particularly in the inpatient setting, it may be challenging to collect comprehensive and transdiagnostic phenotypic data using traditional means.

## Supporting information

S1 Table (DOCX)

S2 Table (DOCX)
