# Supplementary material for: Differences among Research Domain Criteria score trajectories by Diagnostic and Statistical Manual categorical diagnosis during inpatient hospitalization
Source: PLoS One. 2020 Aug 25;15(8):e0237698. doi: 10.1371/journal.pone.0237698 (PMC7447552; doi:10.1371/journal.pone.0237698)
Supplement: S1 Table — (DOCX) [file pone.0237698.s001.docx]

| **Supplemental Table A. Average difference score for each domain and diagnosis with t confidence interval** | | | |
| --- | --- | --- | --- |
| **Diagnosis** | **Domain** | **Change Score** | **[95% Conf. Int]** |
| **Anxiety** | **Arousal & regulatory** | -0.266 | [-0.506 - -0.027] |
| **Anxiety** | **Cognitive** | -0.107 | [-0.455 - 0.241] |
| **Anxiety** | **Negative** | -0.937 | [-1.181 - -0.692] |
| **Anxiety** | **Positive** | -0.624 | [-0.881 - -0.368] |
| **Anxiety** | **Social** | -0.083 | [-0.339 - 0.172] |
|  |  |  |  |
| **BPAD-D** | **Arousal & regulatory** | -0.465 | [-0.618 - -0.313] |
| **BPAD-D** | **Cognitive** | -0.158 | [-0.325 - 0.01] |
| **BPAD-D** | **Negative** | -1.174 | [-1.302 - -1.046] |
| **BPAD-D** | **Positive** | -0.781 | [-0.912 - -0.65] |
| **BPAD-D** | **Social** | -0.15 | [-0.282 - -0.019] |
|  |  |  |  |
| **BPAD-M** | **Arousal & regulatory** | -0.068 | [-0.231 - 0.094] |
| **BPAD-M** | **Cognitive** | -0.126 | [-0.341 - 0.088] |
| **BPAD-M** | **Negative** | -0.726 | [-0.861 - -0.592] |
| **BPAD-M** | **Positive** | -0.632 | [-0.783 - -0.48] |
| **BPAD-M** | **Social** | -0.322 | [-0.514 - -0.13] |
|  |  |  |  |
| **MDD** | **Arousal & regulatory** | -0.357 | [-0.431 - -0.283] |
| **MDD** | **Cognitive** | -0.02 | [-0.098 - 0.059] |
| **MDD** | **Negative** | -1.192 | [-1.254 - -1.13] |
| **MDD** | **Positive** | -0.645 | [-0.713 - -0.577] |
| **MDD** | **Social** | -0.077 | [-0.14 - -0.014] |
|  |  |  |  |
| **Psychosis** | **Arousal & regulatory** | 0.092 | [0.015 - 0.169] |
| **Psychosis** | **Cognitive** | -0.004 | [-0.099 - 0.09] |
| **Psychosis** | **Negative** | -0.748 | [-0.813 - -0.682] |
| **Psychosis** | **Positive** | -0.425 | [-0.495 - -0.355] |
| **Psychosis** | **Social** | -0.199 | [-0.283 - -0.115] |
|  |  |  |  |
| **PTSD** | **Arousal & regulatory** | 0.039 | [-0.287 - 0.366] |
| **PTSD** | **Cognitive** | 0.354 | [-0.038 - 0.745] |
| **PTSD** | **Negative** | -0.955 | [-1.289 - -0.62] |
| **PTSD** | **Positive** | -0.095 | [-0.385 - 0.196] |
| **PTSD** | **Social** | -0.057 | [-0.381 - 0.267] |
|  |  |  |  |
| **Substance** | **Arousal & regulatory** | 0.009 | [-0.239 - 0.258] |
| **Substance** | **Cognitive** | 0.042 | [-0.208 - 0.292] |
| **Substance** | **Negative** | -0.984 | [-1.194 - -0.773] |
| **Substance** | **Positive** | -0.476 | [-0.724 - -0.227] |
| **Substance** | **Social** | -0.188 | [-0.387 - 0.011] |

BPAD-D = bipolar affective disorder, depression
BPAD-M = bipolar affective disorder, mania/mixed
MDD = major depressive disorder
PTSD = post-traumatic stress disorder
