# Supplementary material for: Differences among Research Domain Criteria score trajectories by Diagnostic and Statistical Manual categorical diagnosis during inpatient hospitalization
Source: PLoS One. 2020 Aug 25;15(8):e0237698. doi: 10.1371/journal.pone.0237698 (PMC7447552; doi:10.1371/journal.pone.0237698)
Supplement: S2 Table — (DOCX) [file pone.0237698.s002.docx]

| **Supplemental Table B. Post-hoc pairwise comparisons of RDoC difference scores in domains with significant differences among diagnoses** | | | |
| --- | --- | --- | --- |
|  |  |  |  |
| **Domain / Diagnosis** | **Difference of differences** | **[95% Conf. Int]** |  |
|  |  |  |  |
| **Negative** |  |  |  |
| **BPAD-M - MDD** | 0.466 | [0.224 - 0.707] |  |
| **Psychosis - MDD** | 0.444 | [0.306 - 0.583] |  |
| **BPAD-M - BPAD-D** | 0.447 | [0.161 - 0.734] |  |
| **Psychosis - BPAD-D** | 0.426 | [0.220 - 0.633] |  |
|  |  |  |  |
| **Positive** |  |  |  |
| **Psychosis - MDD** | 0.22 | [0.070 - 0.369] |  |
| **PTSD - MDD** | 0.55 | [0.056 - 1.044] |  |
| **Psychosis - BPAD-D** | 0.356 | [0.133 - 0.580] |  |
| **PTSD - BPAD-D** | 0.687 | [0.166 - 1.208] |  |
|  |  |  |  |
| **Arousal & regulatory** |  |  |  |
| **BPAD-M - MDD** | 0.289 | [0.003 - 0.574] |  |
| **Psychosis - MDD** | 0.449 | [0.286 - 0.613] |  |
| **Substance - MDD** | 0.367 | [0.005 - 0.728] |  |
| **BPAD-M - BPAD-D** | 0.397 | [0.058 - 0.735] |  |
| **Psychosis - BPAD-D** | 0.558 | [0.313 - 0.802] |  |
| **Substance - BPAD-D** | 0.475 | [0.070 - 0.879] |  |

BPAD-M = bipolar affective disorder, mania/mixed
BPAD-D = bipolar affective disorder, depression
MDD = major depressive disorder
PTSD = post-traumatic stress disorder
